# The Effects of Artesunate on the Expression of EGFR and ABCG2 in A549 Human Lung Cancer Cells and a Xenograft Model

**DOI:** 10.3390/molecules161210556

**Published:** 2011-12-19

**Authors:** Hu Ma, Quan Yao, An-Mei Zhang, Sheng Lin, Xin-Xin Wang, Lei Wu, Jian-Guo Sun, Zheng-Tang Chen

**Affiliations:** 1 Cancer Institute of PLA, Xinqiao Hospital, Third Military Medical University, Xinqiao Street No. 2, Chongqing 400037, China; 2 Department of Oncology, Affiliated Hospital of Zunyi Medical College, Zunyi 563000, China

**Keywords:** EGFR, ABCG2, Akt, artesunate, NSCLC, multi-drug resistance

## Abstract

Non-small cell lung cancer (NSCLC) is the leading cause of cancer death worldwide. Clinical and laboratory studies have suggested that multi-targeting approaches against neoplastic cells could help to increase patient survival and might reduce the emergence of cells that are resistant to single-target inhibitors. Artesunate (ART) is one of the most potent and rapidly acting antimalarial agents known, and it also exerts a profound cytotoxic activity toward cancer cells and reverses multi-drug resistance. In the present study, we found that artesunate inhibited NSCLC A549 cell growth and proliferation, induced apoptosis and suppressed tumor growth in a dose-dependent manner in A549 cells and a mouse xenograft model. Furthermore, artesunate down-regulated the expression of epidermal growth factor receptor (EGFR), Akt and ATP-binding cassette subfamily G member 2 (ABCG2) at the mRNA and protein levels *in vitro* and *in vivo*. In conclusion, artesunate is an effective anti-cancer drug that may enhance the effectiveness of other anticancer drugs and may reverse multi-drug resistance by suppressing the transcription of ABCG2, which inhibits drug efflux.

## 1. Introduction

Non-small cell lung cancer (NSCLC) is the leading cause of cancer death worldwide. Despite recent advances in the detection and treatment of lung cancer, the overall five years survival rate remains less than 15% in China. Most patients experience modest clinical benefits from standard platinum-based chemotherapy treatments, which are associated with a limited increase in overall survival [1]. The epidermal growth factor receptor (EGFR) tyrosine kinase inhibitors (TKIs) gefitinib and erlotinib (Tarceva) yield modest increases in survival when administered to NSCLC patients following chemotherapy. Studies have shown that a subset of NSCLC patients treated with these drugs enter remission [2,3]. Notably, this response is correlated with the presence of somatic activating mutations within the EGFR kinase domain [4,5]. However, despite the efficacy of erlotinib and gefitinib in NSCLC patients with EGFR mutations, all patients will ultimately develop resistance to these agents.

The clinical use of EGFR TKIs remains challenging because of drug resistance in tumors, which results in poor prognoses for patients. There is an urgent need to understand the molecular mechanisms of drug resistance and to develop novel therapy strategies. One class of ATP-binding cassette proteins that includes P-glycoprotein, several multidrug resistance proteins, and the ABCG2 protein (also known as BCRP/MXR/ABCP) is suspected to confer drug resistance. These proteins cause multidrug resistance in tumors by actively excluding a wide variety of anticancer drugs from the cell [6,7,8].These proteins are regarded as potential clinical targets for inhibiting multidrug resistance and for altering the absorption, distribution, metabolism, excretion, and toxicity of various chemotherapeutic drugs [9,10]. ABCG2 is a primary active transporter for mitoxantrone, topotecan, and flavopiridol, and its overexpression has been documented in several drug-resistant cell lines and tumors [6,11,12,13]. ABCG2 is expressed in various drug-selected tumor cell lines derived from diverse tissue types, including lung, breast, colon, ovarian and gastric carcinomas, fibrosarcomas and myelomas [14]. ABCG2 has also been shown to bind gefitinib with high affinity, causing the active extrusion of the inhibitor of EGFR and preventing its biological activity [15].

New clinical and experimental studies have suggested that multi-targeting approaches against neoplastic cells could increase patient survival and might reduce the emergence of cells that are resistant to single-target inhibitors [16].

Extracts of *Artemisia annua* have been reported to exhibit various pharmacological activities, such as immunostimulation, anti-inflammation, antitumorogenesis, and antiangiogenesis. One of the major active ingredients in this extract, artesunate (ART), displays anticancer activity *in vivo* and has been successfully used to treat patients with metastatic melanoma [17]. Previous studies have shown that ART may rapidly convert into reactive oxygen species intracellularly, thereby disrupting cellular functions [18]. ART has also been shown to alter principal signaling pathways [19], inhibit metastasis [20], induce DNA damage [21], alter the cell cycle and reverse multidrug resistance [22,23]. However, a definitive mechanism responsible for the cell death and reversal of multidrug resistance associated with ART has yet to be elucidated.

To date, there is limited published data exploring the mechanisms that underlie the efficacy of ART as a combination drug or an inhibitor of drug resistance in treatment regimens, and those existing studies have only examined the interactions of ART with other drugs. Therefore, the aim of this study was to explore the anticancer activity of ART and to investigate the effects of ART on the expression of EGFR and ABCG2 in A549 human lung cancer cells and a mouse xenograft model.

## 2. Results and Discussion

### 2.1. ART Inhibits the Growth and Proliferation of the A549 Cell Line

**Figure 1:**
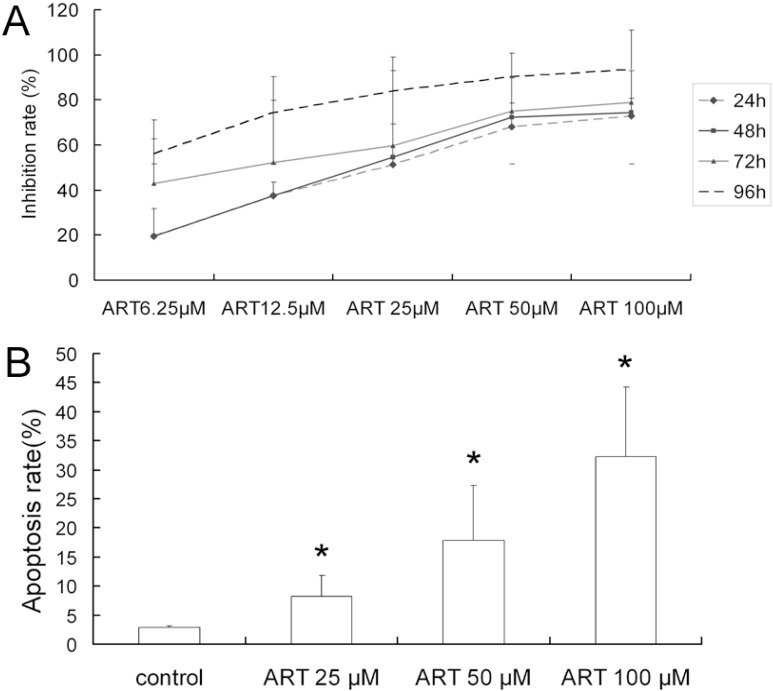
Cytotoxicity of artesunate (ART) in human non-small cell lung cancer cell A549 cells. (**A**) The growth inhibition curves of A549 cells exposed to the indicated concentrations of ART for 24, 48, 72 and 96 h and the mean values from three independent cell viability MTT assays are depicted; (**B**) The percentage of apoptotic cells was determined by flow cytometric analysis following cell staining with Annexin V-FITC and PI. The cells were treated with varying concentrations of ART at 37 °C for 48 h. The data shown represent the means ± SE obtained from three independent experiments. * *p* < 0.05 compared with the control group.

To determine the effect of ART on the growth of A549 cells, the cells were cultured in 96-well plates and treated with ART for 24, 48, 72 and 96 h. The number of viable cells was determined using the MTT assay. The OD values decreased with increased ART concentration and exposure time (*p* < 0.05 compared with the control group). The percentage of inhibition was calculated from the OD value. The results showed that ART has a concentration- and time-dependent inhibitory effect on the growth of A549 cells (Figure 1A).

### 2.2. ART Induces Apoptosis in A549 Cells in a Dose-Dependent Manner

To investigate the mechanism of the cell death induced by ART, flow cytometry analysis was performed on cells treated for 48 h with ART (25–100 μM). The percentages of apoptotic A549 cells increased with increasing ART concentrations: 8.30% ± 3.45% of cells were apoptotic following treatment with 25 μM ART, compared with 32.3% ± 11.98% after 100 μM ART (Figure 1B).

### 2.3. The Effects of ART on ABCG2 and EGFR mRNA Levels in Lung Cancer Cells

**Figure 2:**
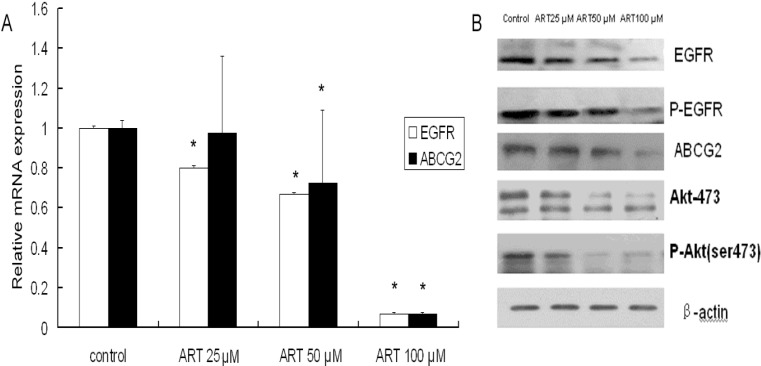
ART down-regulates the EGFR and ABCG2 mRNA and protein levels in A549 cells. (**A**) Cells were treated with varying concentrations of ART for 48 h and then harvested. The EGFR and ABCG2 mRNA levels were detected using real-time RT-PCR; (**B**) The expression of EGFR, p-EGFR Akt, p-Akt and ABCG2 was measured by western blot analysis. A549 cells were treated with varying concentrations of ART for 48 h, then β-actin expression was used as an internal control to determine the expression levels of proteins. Significant down-regulation of all proteins was observed. The blots are representative of three independent experiments. The data represent the means ± SE from three independent experiments performed in triplicate. * *p* < 0.05 compared with the control group.

A549 cells were treated with 25–100 μM ART for 48 h, washed, and analyzed for the mRNA expression of EGFR and ABCG2 using real-time RT-PCR. The mRNA expression of EGFR was slightly down-regulated when the A549 cells were treated for 48 h with low and medium concentrations of ART (25 and 50 μM). When the concentration of ART was increased to 100 μM, however, the expression of EGFR was significantly down-regulated compared with that of the controls (*p* < 0.05). In contrast, a down-regulation of ABCG2 mRNA expression was observed with medium and high concentrations of ART (50 and 100 μM) (*p* < 0.05), although a low concentration of ART (25 μM) did not affect the expression (Figure 2A).

### 2.4. The Effects of ART on EGFR, Akt and ABCG2 Protein Levels in Lung Cancer Cells

To determine whether cell apoptosis is related to the inhibition of important cell growth signaling pathways, a Western blot analysis was performed. Because A549 cells express EGFR, we determined the phosphorylation status of EGFR after a 48-h exposure to ART. ART decreased EGFR, EGFR phosphorylation and prevented the activation of the downstream kinase Akt. The phosphorylation of Akt and ABCG2 decreased in a concentration-dependent manner (Figure 2B). The above results indicate that the inhibition of cell growth may be related to the inhibition of pro-survival signals in A549 cells. The synergistic effect of the drug may be explained by the inhibition of ABCG2 expression, which would prevent the cellular efflux of the drug by ABCG2.

### 2.5. ART Suppresses Tumor Growth in a Mouse Xenograft Tumor Model

**Figure 3:**
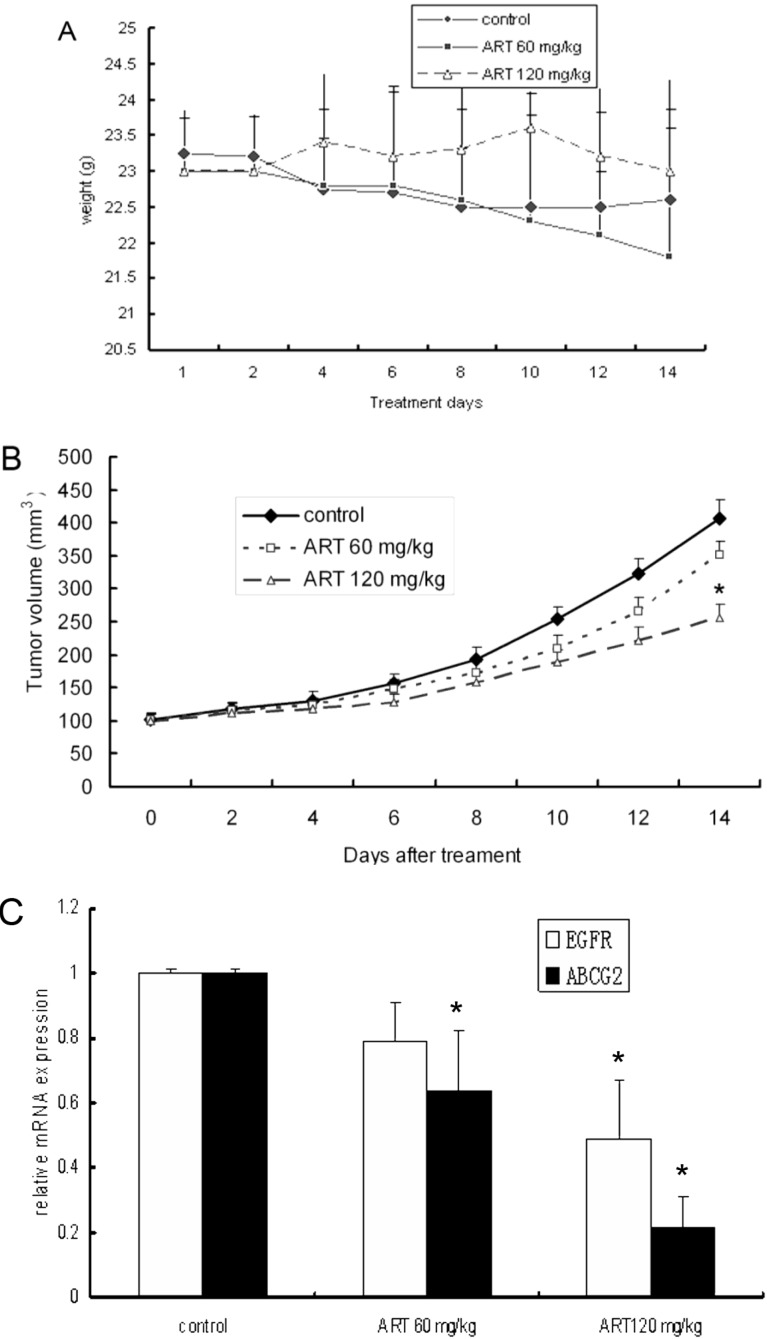
ART suppresses tumor growth and EGFR and ABCG2 mRNA levels in A549 xenografts. (**A**) The effect of ART on the mean tumor volume in A549 human non-small cell lung cancer xenograft mice was explored. The mice were treated daily with oral doses of ART at 0, 60 and 120 mg·kg^−1^ for 14 days. The tumor size was measured 3 times per week. The volumes presented are the means from 5 mice; (**B**) The body weights of the ART-treated mice were similar to that of saline controls; (**C**) The expression of EGFR and ABCG2 mRNA was measured in A549 xenografts. The mice were administered daily oral doses of 60 mg·kg^−1^ ART, 120 mg·kg^−1^ ART or distilled water. The EGFR and ABCG2 mRNA expression levels were measured using real-time RT-PCR. * p < 0.05 compared with the control group.

To determine whether ART has potential therapeutic value for lung carcinoma treatment, we further tested the inhibition of A549 cells by ART in an athymic mouse xenograft model. A549 cells (1 × 10^6^ cells per mouse) were injected subcutaneously into mice in three groups: a distilled water control group and two ART groups (60 mg·kg^−1^ or 120 mg·kg^−1^, n = 5). Caliper measurements of the longest perpendicular tumor diameters were performed every other day to estimate the tumor volume. The results showed that 120 mg·kg^−1^ ART significantly suppressed (*P <* 0.05) tumor growth (Figure 3A). These findings demonstrate that ART potently suppresses lung tumor growth *in vivo*.

### 2.6. ART Suppresses EGFR, Akt and ABCG2 Protein and Gene Expression in Vivo

**Figure 4:**
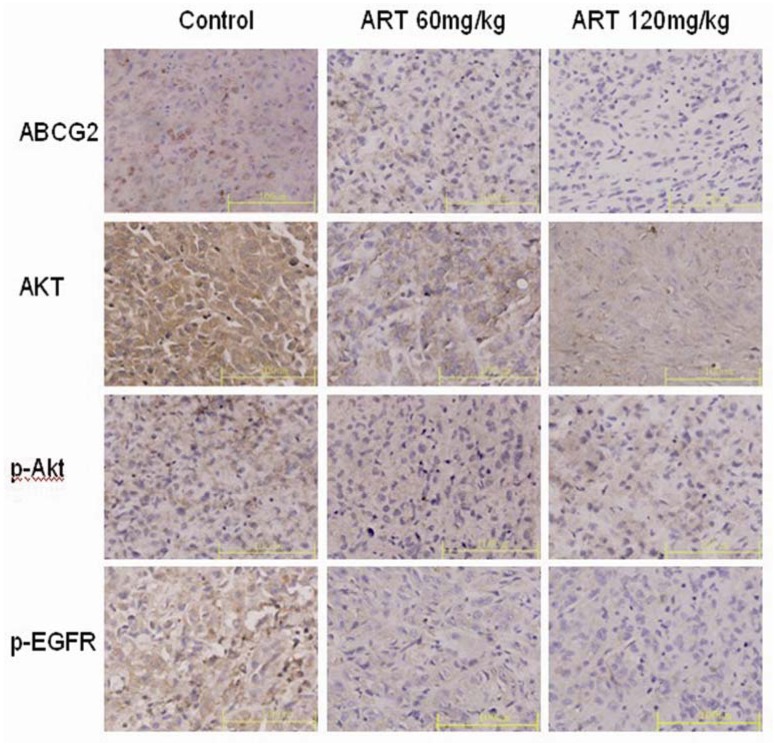
The expression of ABCG2, Akt, p-Akt and p-EGFR proteins was examined in untreated and ART-treated (60 mg·kg^−1^ and 120 mg·kg^−1^) xenografts. Photographs of immunohistochemical staining for ABCG2, Akt, p-Akt and p-EGFR (×40) are shown. A distinct yellow stain can be observed in the membrane and cytoplasm. The images presented are representative of three independent experiments. The data are the means ± SE from three independent experiments performed in triplicate. * p < 0.05 compared with the control group. Bars, 100 μm.

Further investigation revealed an *in vivo* suppression of EGFR and ABCG2 protein and gene expression by oral artesunate. The mRNA expression of EGFR was slightly down-regulated in mice treated with 60 mg·kg^−1^ ART compared with the controls (*p* > 0.05). However, the mRNA expression of EGFR was significantly down-regulated in the 120 mg·kg^−1^ ART group mice compared with the controls (*p* < 0.05). In contrast, a down-regulation of ABCG2 mRNA expression was observed with both doses of ART (60 and 120 mg·kg^−1^) (*p* < 0.05 compared with the controls) (Figure 3C). In all the tumor tissue slices, the cytoplasm and membranes of the control cells had more robust staining than was observed in the samples treated with ART. Distinct staining was observed in each group, but in contrast to the control xenograft, little or no staining for p-EGFR or ABCG2 was observed in the 120 mg·kg^−1^ ART treatment group (Figure 4).

### 2.7. Discussion

EGFR is a member of the erbB/HER family of receptor tyrosine kinases (TKs). Upon binding to its ligands, EGFR homodimerizes or heterodimerizes with other erbB receptors, resulting in the autophosphorylation of the receptor at specific TK residues within the cytoplasmic tail. The activated receptor recruits signaling complexes and activates the Ras/mitogen-activated protein kinase (MAPK), extracellular signal-regulated kinase (ERK), phosphatidylinositol 3-kinase (PI3K)/Akt, signal transducer and activator of transcription (STAT), and phospholipase C gamma pathways. These pathways are potent oncogenic regulators of tumor cell growth, invasion, transformation, and survival. EGFR has also been implicated in angiogenesis, invasion via matrix metalloproteinases, and tumor cell motility, all of which contribute to metastasis [24,25,26]. EGFR is frequently overexpressed in human tumors such as NSCLC, head and neck squamous cell carcinoma (HNSCC), and glioblastoma [27].

The present study shows that ART inhibits the growth and proliferation of A549 cells in a concentration- and time-dependent manner and induces apoptosis in a dose-dependent manner. After treatment with ART, the expression of EGFR was down-regulated in A549 cells and in the xenograft tumor model. These findings suggest that EGFR may be involved in ART-mediated cytotoxicity and may be a promising target in ART treatment of lung cancer.

The efficacy of cancer chemotherapy is limited by cellular mechanisms of resistance that result in the increased efflux of chemotherapeutic agents, thereby reducing intracellular drug levels. The ability of cells to acquire resistance to multiple compounds, termed multidrug resistance (MDR), is often mediated by the overexpression of ATP-binding cassette (ABC) transporters, which move substrates out of the cell against a concentration gradient [28]. Members of the ATP-binding cassette transporter family have been associated with resistance to chemotherapy and ionizing radiation in several malignancies, such as leukemia, melanoma, and breast, pancreatic, and colorectal cancers. Studies evaluating cells with the SP phenotype have shown that stem cells overexpress ABCG2 but not ABCB1, the transporter targeted in most clinical studies [29], therefore, it is important to develop inhibitors for ABCG2. The compound fumitremorgin C (FTC) is a natural product that specifically inhibits ABCG2 [30]. However, this compound is toxic to both cells and mice, rendering it unsuitable for clinical studies.

To date, several BRCP/ABCG2 inhibitors with different structures have been reported, but the exact mechanisms of inhibition have not been sufficiently investigated [30]. Cytokines and growth factors have also been reported to alter the expression of the ABCG2 gene. When ABCG2-positive side population cells were isolated from MCF-7 cells and treated with transforming growth factor-beta, ABCG2 gene expression decreased [31]. Treatment of primary term trophoblasts with tumor necrosis factor-alpha or interleukin-1 beta decreased the mRNA and protein levels of ABCG2 whereas treatment with insulin-like growth factor II increased expression [32]. Further studies are warranted to accurately characterize the mechanisms that control ABCG2 expression. Several studies have reported that the protein kinase Akt is involved in regulating the surface expression of the ABCG2 protein. Mogi *et al*. were the first to report that Akt1-deficient mice displayed a reduced number of side population cells [33], a distinct population of Abcg2-positive hematopoietic stem cells that become visible when bone marrow cells are incubated with the DNA dye Hoechst 33342. When side population cells from normal mice were incubated with the phosphatidylinositol 3-kinase inhibitor LY294002, Abcg2 was translocated from the plasma membrane to the cytoplasm, although the total protein expression did not appear to be affected [33]. When bone marrow cells were transfected with Akt1, an increased number of cells was observed in the side population [34]. Takada *et al*. subsequently reported that in ABCG2-transfected LLC-PK1 polarized kidney cells, the phosphatidylinositol 3-kinase inhibitors LY294002 and wortmanin both caused a shift of ABCG2 expression from the apical membrane to the intracellular space and that the shift correlated with the phosphorylation state of Akt [14]. In the present study, we found that the treatment of A549 cells and a xenograft tumor model with ART inhibited the phosphorylation of Akt and significantly down-regulated the mRNA and protein levels of ABCG2. These results imply that ART may regulate the expression of ABCG2 in lung cancer cells *in vitro* and *in vivo*. ART may down-regulate the EGFR downstream signal via PI3K/Akt. It has been reported that ART does not inhibit ABCG2 function for pumping drugs out of cells [35], however, the present study demonstrated that ART down-regulates the mRNA and protein levels of ABCG2. Thus, we hypothesize that low concentrations of ART do not inhibit ABCG2 function, but high levels may. This theory needs to be tested in future studies.

## 3. Experimental

### 3.1. Reagents

ART was obtained from Sigma Aldrich (CA). Rabbit polyclonal antibodies to EGFR and p-EGFR (phospho S1070) and a rat monoclonal antibody (Bxp-53) to BCRP/ABCG2 were purchased from Abcam Hong Kong Ltd. (Hong Kong, China). Rabbit polyclonal antibodies to Akt (Ab-473) and p-Akt (Phospho-Ser473) were purchased from Signalway Antibody Co., Ltd (Pearland, TX, USA), and goat anti-rabbit IgG (peroxidase conjugate) and goat anti-rat IgG (peroxidase conjugate) were purchased from Boster (Wuhan, China). Methylthiazolyltetrazolium (MTT) was obtained from Sigma Aldrich.

### 3.2. Cells and Treatments

A549 human lung cancer cells were purchased from the American Type Culture Collection (Rockville, MD, USA). The A549 cells were maintained in RPMI-1640 medium supplemented with 10% fetal bovine serum, 100 U/mL penicillin and 100 μg/mL streptomycin (all reagents were purchased from HyClone, Thermo, China) in a humidified atmosphere containing 5% CO_2_ at 37 °C. The A549 cells were seeded in bottles at 5 × 10^5^ cells per bottle and cultured for 24 h. The cells were then treated with ART at 12.5–100 μM for 48 h. The ART was dissolved in dimethylsulfoxide (DMSO, Sigma) as a stock solution (100 mM) and added directly to the medium. Following treatment, the cells were harvested from the substrate using 0.05% trypsin and 0.02% EDTA and washed twice with phosphate-buffered saline (PBS). The treated cells were used for RNA and protein extraction.

### 3.3. Proliferation Assays

Exponentially growing cells were added to 96-well plates at a density of 5 × 10^3^/well. ART (6.25–100 μM) was then added to the wells, with a total volume of 200 μL in each well. The cell number was measured at 24, 48, 72 and 96 h using a standard methylthiazolyltetrazolium (MTT)-based assay. Briefly, MTT was added to each well at a working concentration of 1 mg/mL, and the plates were returned to the incubator for 4 h. After this time, the medium was removed by aspiration, 150 μL DMSO was added to each well and the plates were gently agitated for 10 min before measuring the optical density at 490 nm. The effect of the treatment was determined as the percentage of viability compared with the untreated cells.

### 3.4. Apoptosis

A549 cells (2 × 10^5^) were cultured for 48 h and treated with the ART (25, 50 and 100 μM) in triplicate. The cells were stained by dual-color fluorescence with Annexin V/fluorescein isothiocyanate (FITC) and propidium iodide (Beyotin Biological Engineering Co. Ltd., China). After incubating 3 × 10^4^ cells for 15 min in the dark, the apoptotic cells were counted by flow cytometry using an EPICS XL flow cytometer (Beckman Coulter).

### 3.5. Animal Experiments

Five-week-old male BALB/c athymic nude mice were obtained from the Animal Research Center, Xinqiao Hospital, Third Military Medical University, China. All the animal experiments complied with the WHO guidelines for the humane use and care of animals. Tumors were established by the subcutaneous injection of 1 × 10^6^ A549 cells into the right armpits of the mice. The mice were inspected for tumor formation twice per week, and the tumor size was measured using a slide caliper. The tumor volume (V) was calculated according to the empirical equation V = (length × width^2^)/2. When the tumors reached a mean volume of approximately 100 mm^3^, the mice were randomly assigned to three groups (n = 5) and administered the following treatments: (**a**) distilled water (control); (**b**) 60 mg·kg^−1^ ART or (**c**) 120 mg·kg^−1^ ART. Each animal received the treatment through gavage once daily for two weeks. The mice were closely monitored before the tumors were removed. Each tumor was split into three pieces: One was fixed in 10% paraformaldehyde, one was stored in RNAiso Reagent at −80 °C for later real-time RT-PCR analysis and the third was stored at −80 °C for further analysis.

### 3.6. Real-time RT-PCR Analysis

Real-time RT-PCR was used to determine the transcript levels of EGFR and ABCG2 in A549 cells and lung cancer xenografts with the RNAiso Reagent and the SYBR^®^ PrimeScrip^®^ RT-PCR Kit (TaKaRa Biotechnology, Dalian, China). The primer sequences for the target genes can be found in Table 1. The relative differences in expression between groups were expressed using cycle time (Ct) values as follows: The Ct values of the genes of interest were first normalized against β-actin from the same sample, and then the relative differences between the control and treatment groups were calculated and expressed as relative increases or decreases, with the control on the Applied Biosystems 7500 system set to 1.0.

**Table 1:** Primer sequences for real-time RT-PCR analysis.

	GenBank		
Gene	Accession#	Forward	Reverse
β-actin	NM_-_001101.3	GTGAAGGTGACAGCAGTCGGTT	GAAGTGGGGTGGCTTTTAGGA
EGFR	NM_-_005228.3	TTTGGGAGTTGATGACCTTTGG	ACGGAACTTTGGGCGACTATCT
ABCG2	NM_031512	TGAAACCTGGTCTCAACGCCATCC	CGTCAGAGTGCCCATCACAACAT

### 3.7. Immunohistochemical Staining

Tumor samples were fixed in 10% paraformaldehyde and embedded in paraffin. Sections for immunohistochemistry were cut at 4 μm. For EGFR, P-EGFR and ABCG2 detection, antigen retrieval was performed by immersing sections in Target Retrieval Solution pH 6.0 (DakoCytomation, Carpinteria, CA, USA) and then heating them in a microwave oven for 20 min. Endogenous peroxidase activity was quenched by incubation in 3% H_2_O_2_ for 15 min. To block non-specific binding sites, the sections were treated with 5% bovine serum albumin (BSA) for 20 min. The sections were incubated with primary antibodies overnight at 4 °C (1:100 dilutions for EGFR and P-EGFR and 1:50 for ABCG2). The rabbit polyclonal antibodies were detected using a peroxidase-conjugated goat anti-rabbit IgG secondary antibody for EGFR and p-EGFR, as well as a peroxidase-conjugated rabbit anti-rat IgG secondary antibody for ABCG2. DAB was used for staining, and BSA was used to replace the primary antibodies in the control. PBS was used to replace the antibodies in the working control group. The sections were counterstained with hematoxylin.

### 3.8. Western Blotting

For Western blotting analysis, 40 μg of total protein was resolved by SDS polyacrylamide gel (Bio-Rad) electrophoresis, and the proteins were then transferred onto polyvinylidene difluoride membranes (Bio-Rad). After four washes, the membranes were incubated with the Blocking kit (Boster, China) for 1 h at room temperature and then incubated overnight at 4 °C with the following primary antibodies: Anti-EGFR, anti-phospho-EGFR (p-S1070, Abcam, Hong Kong), anti-Akt and anti-phospho-Akt (p-Ser473) (1:1,000 dilution, Signalway Antibody). The protein levels were normalized against β-actin from the same sample (1:3,000 dilution, Boster, China). After three washes, the membranes were incubated for 1 h at room temperature with species-specific horseradish peroxidase-conjugated secondary antibodies. The membranes were developed using an enhanced chemiluminescence (ECL) detection system followed by exposure to ECL-Hyperfilm (Beyotain, China).

### 3.9. Statistical Analysis

All the assays were repeated three times, and the results are expressed as the means ± SD. The quantitative experiments were analyzed using Student’s *t*-test. SPSS 17.0 statistical software (SPSS Inc., Chicago, IL, USA) was used, and *p <* 0.05 was considered statistically significant.

## 4. Conclusions

In summary, our results show that ART exerts antitumor effects through the down-regulation of EGFR and its downstream factor Akt in lung cancer cells. Furthermore, ART down-regulates the expression of ABCG2 *in vitro* and *in vivo*. Because of the diversity of its targets, ART could be used in combination with other agents that act reciprocally [36,37]. Furthermore, the efficacy of ART could be enhanced by combining it with other drugs to reverse the multi-drug resistance mediated by ABCG2. Taken together, the results of this study indicate that ART possesses significant antitumor activity and represents a potential treatment for non-small cell lung cancer.
